# Noradrenaline from Locus Coeruleus Neurons Acts on Pedunculo-Pontine Neurons to Prevent REM Sleep and Induces Its Loss-Associated Effects in Rats

**DOI:** 10.1523/ENEURO.0108-16.2016

**Published:** 2016-12-08

**Authors:** Mudasir Ahmad Khanday, Bindu I. Somarajan, Rachna Mehta, Birendra Nath Mallick

**Affiliations:** School of Life Sciences, Jawaharlal Nehru University, New Delhi 110607, India

**Keywords:** inhibition, REM-OFF/REM-ON neurons, REMS deprivation, REMS generation, RNAi, tyrosine hydroxylase

## Abstract

Normally, rapid eye movement sleep (REMS) does not appear during waking or non-REMS. Isolated, independent studies showed that elevated noradrenaline (NA) levels inhibit REMS and induce REMS loss-associated cytomolecular, cytomorphological, psychosomatic changes and associated symptoms. However, the source of NA and its target in the brain for REMS regulation and function in health and diseases remained to be confirmed *in vivo*. Using tyrosine hydroxylase (TH)-siRNA and virus-coated TH-shRNA in normal freely moving rats, we downregulated NA synthesis in locus coeruleus (LC) REM-OFF neurons *in vivo*. These TH-downregulated rats showed increased REMS, which was prevented by infusing NA into the pedunculo-pontine tegmentum (PPT), the site of REM-ON neurons, normal REMS returned after recovery. Moreover, unlike normal or control-siRNA- or shRNA-injected rats, upon REMS deprivation (REMSD) TH-downregulated rat brains did not show elevated Na-K ATPase (molecular changes) expression and activity. To the best of our knowledge, these are the first *in vivo* findings in an animal model confirming that NA from the LC REM-OFF neurons (1) acts on the PPT REM-ON neurons to prevent appearance of REMS, and (2) are responsible for inducing REMSD-associated molecular changes and symptoms. These observations clearly show neuro-physio-chemical mechanism of why normally REMS does not appear during waking. Also, that LC neurons are the primary source of NA, which in turn causes some, if not many, REMSD-associated symptoms and behavioral changes. The findings are proof-of-principle for the first time and hold potential to be exploited for confirmation toward treating REMS disorder and amelioration of REMS loss-associated symptoms in patients.

## Visual Abstract

**Figure:**
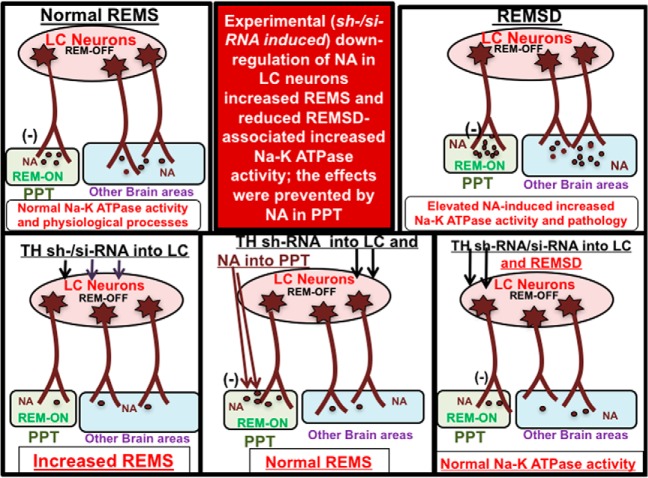

## Significance Statement

Reciprocal interactions among rapid eye movement (REM)-ON and REM-OFF neurons in pedunculo-pontine tegmentum (PPT) and locus coeruleus (LC), respectively, have been proposed to regulate REM sleep (REMS). Findings from isolated independent studies led to the proposition that noradrenaline (NA) from the latter inhibits the former to prevent REMS, and that its withdrawal initiates REMS by disinhibiting the former, while excess NA causes REMS loss-associated symptoms. However, evidence from direct *in vivo* studies confirming this idea was lacking. Using RNAi technology *in vivo,* we show that NA from LC neurons prevents REMS by inhibiting PPT neurons and that those LC neurons are the source of NA for inducing REMS loss-associated symptoms. These confirmatory findings in animal models are the first proof-of-principle that holds the potential for exploitation in ameliorating REMS loss-associated symptoms in patients.

## Introduction

Rapid eye movement sleep (REMS) is a unique cognitive state expressed at least in animals higher in evolution, including humans. Its duration varies among species, is maximally expressed early in development, and reduces with aging; however, it is never absent through life (Roffwarg et al., 1966). Normally, REMS does not appear during waking but appears only after a period of non-REMS (NREMS). Interestingly, among the classic known conscious states, although one spends the least amount of time in REMS per day, it is affected in almost all psychosomatic–behavioral disorders (Bliwise et al., 1990; Rechtschaffen et al., 2002; Mallick et al., 2005; Comella, 2007; Mallick and Singh, 2011). Experimental REMS deprivation (REMSD) has been reported to affect several physiological processes, including thermoregulation (Salin-Pascual et al., 1997; Jaiswal and Mallick, 2009), cardiovascular–respiratory systems (Everson et al., 1989), metabolism (Thakkar and Mallick, 1993b; Koban and Swinson, 2005), memory consolidation (Stickgold and Walker, 2007), brain development and maturation (Marks et al.,1995), and excitability (Mallick and Singh, 2011; Placidi et al., 2013; Amar et al., 2016). Prolonged REMSD has been reported to have fatal consequences (Kushida et al., 1989). We posited that REMS serves fundamental physiological processes and housekeeping functions of the brain (Mallick and Singh, 2011). Although it serves such fundamental physiological processes, our knowledge about the precise mechanism of neural regulation of REMS is incomplete. We argued that REMS regulation will be multifactorial and that various factors that affect it would modulate the basic scaffold neurons in specific neuronal circuitry responsible for its regulation. Further, we also proposed that if the symptoms and effects associated with REMS disturbance were specific to REMS loss, those changes would, by and large, be modulated by a common factor (neurotransmitter), which should be a part of the same basic scaffold neuronal circuitry and must be crucial for REMS regulation.

It has been well established that during REMS, REM-ON neurons in the pedunculo-pontine tegmentum (PPT) increase firing, while the noradrenaline (NA)-ergic REM-OFF neurons in the locus coeruleus (LC) cease firing; however, their causal relationship, if any, was unknown. Interaction among those REM-ON and REM-OFF neurons forms the basic scaffold for REMS regulation (Hobson et al., 1975; Jacobs, 1986; Mallick et al., 2012). It was proposed that during NREMS the NAergic REM-OFF LC neurons possibly inhibit the REM-ON neurons in the PPT; the LC-REM-OFF neurons must cease firing for the generation of REMS; and, if NAergic REM-OFF neurons are not allowed to cease activity, REMS would not appear, leading to disturbed REMS (Singh and Mallick, 1996; Mallick et al., 2001, 2012). Furthermore, it was also proposed that, if NAergic REM-OFF neurons do not cease activity, the NA level would rise in the brain and that elevated NA levels would be responsible for REMS loss-associated effects and symptoms. Indeed, our contention was supported by indirect, independent, and isolated experimental studies as well as by clinical observations (Mallick and Singh, 2011; Gannon et al., 2015); however, direct evidence particularly from *in vivo* studies was lacking. Therefore, in this study using tyrosine hydroxylase (TH)-siRNA and TH-shRNA in a separate group of normal rats, we downregulated TH in the LC neurons inhibiting NA synthesis in REM-OFF neurons. REMS was estimated in those TH-downregulated rats with or without infusing NA into the PPT, the site of REM-ON neurons. Separate sets of control (Ctrl) and conditional TH-downregulated (which would downregulate NA) rats were deprived of REMS, and Na-K ATPase activity was estimated in the brains of those rats. We observed that normally NA released from the LC neurons inhibits the PPT REM-ON neurons to prevent the appearance of REMS. Additionally, in NA-downregulated REMS-deprived rats, REMSD-associated molecular changes, which were otherwise expressed in the brains of normal REMS-deprived rats, were not expressed. These *in vivo* results are from rats in which most other REMS and REMS loss-related studies have been conducted. To the best of our knowledge, these findings are the first direct evidence from an *in vivo* study. The results are proof-of-principle and hold potential to be exploited as a therapy to ameliorate REMS loss-associated symptoms including in humans.

## Materials and Methods

### Animals

Sixty-one healthy adult male Wistar rats (250–330g), divided into 11 groups, obtained from our university animal facility were used (Table 1). The rats were housed at 23 ± 1°C and 30–70% humidity under 12 h light/dark cycle with *ad libitum* access to food and water. National Institutes of Health guidelines for care and use of laboratory animals were followed, and all of the experimental protocols were approved by the Institutional Animal Ethics Committee of Jawaharlal Nehru University. All efforts were made to use the minimum number of animals to obtain a meaningful, statistically valid result and to minimize discomfort, pain, and suffering in the rats.

**Table 1:** Experimental protocol used in the study

Groups	Stereotaxic surgery	Experimental procedure		
Group I (*n* = 5)	Microinjection of Ctrl-shRNA and TH-shRNA into the left and right LC, respectively, during surgery	Postsurgical recovery for 8 d	8 d after injection, LC punched out for *TH* mRNA estimation by qRT-PCR	
Group II (*n* = 4)			8 d after injection LC punched out for TH protein estimation by Western blot	
Group III (*n* = 5)			8 d after injection 40 μm brain sections through LC were immunostained with TH and positive neurons counted	
Group IV (*n* = 6)	Microinjection of TH-shRNA bilaterally into LC during surgery and implantation of cannula bilaterally into PPT		8 d after TH-shRNA injection into LC, on day 1 saline was injected bilaterally into PPT and sleep wakefulness recorded for 12 h.On day 2, NA was injected into PPT and sleep–wakefulness recorded in the same rat for 12 h.3 weeks after TH-shRNA injection, sleep wakefulness was recorded for 12 h	
Group V (*n* = 5)	Microinjection of Ctrl-shRNA bilaterally into LC during surgery		8 d postsurgery injection sleep–wakefulness recording was done for 12 h	
Group VI (*n* = 6)	Implantation of cannula bilaterally into LC		After postsurgical recovery, first day either Ctrl-siRNA or TH-siRNA was microinjected bilaterally into LC and sleep–wakefulness recorded next day for 12 h.After 7 d Ctrl-siRNA (which was not injected on the first day) was injected and recording continued.	
Group VII (*n* = 10)	FMC		FMC home cage without any microinjection	Whole-brain homogenate was prepared and(1) Na-K ATPase activity was estimated(2) TH protein estimation by Western blot
Group VIII (*n* = 5)	Implantation of cannula into LC bilaterally		REMSD for 96 h with microinjection of Ctrl-siRNA at 24 and 48 h	Whole-brain homogenate was prepared, and Na-K ATPase activity was estimated
Group IX (*n* = 5)	Implantation of cannula into LC bilaterally		REMSD for 96 h with microinjection of TH-siRNA at 24 and 48 h	Whole-brain homogenate was prepared and Na-K ATPase activity was estimated
Group X (*n* = 5)	Microinjection of Ctrl-shRNA during surgery into LC bilaterally		After recovery REMSD for 96 h	Whole-brain homogenate was prepared and(1) Na-K ATPase activity was estimated(2) Western blot analysis of TH protein was performed
Group XI (*n* = 5)	Microinjection of TH-shRNA during surgery bilaterally into LC		After recovery REMSD for 96 h	Whole-brain homogenate was prepared and(1) Na-K ATPase activity was estimated and(2) Western blot analysis of TH protein was performed
Total no. of Rats	61			

### Stereotaxic surgery in rats

Under isoflurane (Baxter Healthcare)-induced surgical anesthesia using a brain atlas (Paxinos and Watson, 1998), all rats underwent stereotaxic surgery (Table 1) for either or a combination of (1) implantation of electrodes for sleep–waking recording; (2) short-term (during surgery) microinjection of TH-shRNA or Ctrl-shRNA bilaterally into the LC; and (3) implantation of self-designed and self-fabricated guide cannulae with obturators (blockers) bilaterally either into the LC or PPT. Rats in groups IV, V, and VI were implanted with self-designed and self-fabricated sterile electrodes for bipolar electroencephalogram (EEG), electromyogram (EMG), and electrooculogram (EOG) recordings. A ground electrode was also implanted on the midline above the frontal sinus. The free ends of the EEG, EOG, and EMG electrodes and the ground electrode were soldered to a 9-pin female plug, which was fixed on the skull with dental acrylic cement. The surgical procedures, including the implantation of cannulae, were completed in accordance with previous reports (Singh and Mallick, 1996; Mallick et al., 2001; Khanday and Mallick, 2015). After surgery, the rats were allowed to recover for at least a week with adequate postoperative care. During the recovery period from surgical trauma, the rats were acclimatized to the recording environment, experimenter, handling, recording chamber, and recording cables prior to data collection. To habituate rats for microinjections, mock injection sessions were also performed during this recovery period.

### Microinjection

The details of the group-wise microinjection of chemicals and the experiments conducted have been summarized in Table 1. For microinjection, all working solutions at desired concentrations were freshly prepared. For short-term injection during surgery using a Quintessential Stereotaxic Injector fitted with a 1 μl Hamilton syringe, 0.5 μl of either TH-shRNA or Ctrl-shRNA was stereotaxically microinjected bilaterally into the LC at the rate of 0.05 μl/min. In chronically prepared, freely behaving rats, microinjections were made using a 33 ga (Plastic One) injector cannula connected to a 1 μl Hamilton syringe. The injector was connected to the Hamilton syringe with tightly fitting flexible polythelene tubing (∼10 cm). The injector cannula had a stopper arrangement so that, when it was introduced inside the brain through the guide cannulae, the tip of the injector projected out by 1 mm from the tip of the guide cannulae to reach about the middle of the desired target area in the brain, the LC, or the PPT (as the case may be).

### Long-term recording

Electrophysiological parameters signifying sleep–wakefulness were recorded from the chronically prepared, freely behaving rats. The recording was performed in a semi-soundproof Faraday cage cubicle, which was fitted with a commutator. To avoid entangling causing discomfort to the rats, the recording wires were connected to the commutator. Video monitoring displayed the behavior of the recording rats in real time on a remote monitor outside the cubicle without disturbing them.

### REMS deprivation

Rats were deprived of REMS for 96 h using the classic flowerpot method (Jouvet-Mounier et al., 1965; Gulyani and Mallick, 1993). In brief, the rats were maintained on a small platform (6.5 cm) raised over surrounding water in a tank. The rats could sit and move around freely on the platform and had free access to food and water. Platform size was selected based on the body weight of the rats, as reported previously (Yanik and Radulovacki, 1987); we have been using this method for a long time. In this method, although the animals are deprived initially of both NREMS and REMS, beyond 48 h on the small platform, the rats are primarily deprived of REMS (Mendelson et al., 1974; McDermott et al., 2003; we had also confirmed REMSD (unpublished data) in this laboratory while standardization of the method).

### TH-siRNA, TH-shRNA, and NA and their delivery

Lentiviral-coated TH-shRNA or its Ctrl-shRNA or TH-siRNA sequences were customized and purchased from Sigma-Aldrich; Ctrl-siRNA was purchased from Ambion (Table 2). In a separate set of experiments, rats were microinjected (TH-shRNA, TH-siRNA, or controls) bilaterally in the LC to inhibit the TH in NAergic LC neurons to downregulate NA synthesis, while NA (Sigma-Aldrich) was microinjected bilaterally into the PPT.

**Table 2:** TH-siRNA, Ctrl-siRNA, TH-shRNA, and Ctrl-shRNA sequence, primers

Name	Sequence (accession number)
TH-siRNA	Sense strand- 5'CUGUGAAGUUUGACCCGUAtt-3' (NM_012740)
	Antisense strand-5'UACGGGUCAAACUUCACAGgg-3' (NM_012740)
Ctrl-siRNA	Non-targeting control (Ambion, 4390843)
TH-shRNA	5'CCGGCAGGAACTATGCCTCTCGTATCTCGAGATA CGAGAGGCATAGTTCCTGTTTTTG 3' (TRCN0000115525 NM_009377.1-1340s1c1)
Ctrl-shRNA	Turbo GFP shRNA control particles (SHC004V)
*TH*-Forward primer	5'GTATATCCGCCATGCCTCCT3' NM_012740.3
*TH*-Revers primer	5'AATGTCCTGGGAGAACTGGG3' NM_012740.3
*TBP*-Forward primer	5'ACCTAAAGACCATTGCACTTCG3' NM_001004198
*TBP*-Reverse primer	5'GCTCTCTTATTCTCATGATGACTGC3' NM_001004198
*GAPDH*-Forward primer	5'AGGTCGGTGTGAACGGATTTG3' NM_017008
*GAPDH*-Reverse primer	5'TGTAGACCATGTAGTTGAGGTCA3' NM_017008

### Experimental design

#### Experiment 1: gene knockdown studies

During surgery, rats in groups I, II, and III received microinjections of either TH-shRNA or Ctrl-shRNA into one side each (right or left) of the LC. After 8 d, rats in groups I and II were guillotined and brains were removed, followed by punching out the left and right LCs separately (Mehta et al., 2015). Using qRT-PCR, TH mRNA was estimated in those punched regions of group I rats (Amar and Mallick, 2015), while TH-protein was quantified using Western blotting in the punched LCs of group II rats. Brains of group III rats were perfused, and the brainstem containing both (left and right side) LCs was sectioned at 40 µm using a cryostat (Leica, Germany) and immunostained (Jaiswal et al., 2010) using TH-antibodies (catalog #AB152, Millipore; RRID:AB_390204).

##### Real-time PCR

***RNA isolation and reverse transcription.*** Total RNA from right and left LCs was separately isolated using TRIzol reagent (Ambion). Isolated RNA was aliquoted, quantified using a nanodrop, and stored at −80^°^C until further use. A total of 1 µg of RNA was reverse transcribed using the First-Strand cDNA Synthesis Kit (Fermentas) following the manufacturer instructions. The real-time primers of *TH*, glyceraldehyde-3-phosphate dehydrogenase (*GAPDH*), and TATA binding protein (*TBP*) were designed using Primer 3 software and the NCBI tool (Table 2). The cDNA (prepared from an equal amount of RNA) of Ctrl-shRNA and TH-shRNA were subjected to quantification using the ABI Prism 7500 FAST Real-Time PCR system (Applied Biosystems). Melting peaks were determined using melting curve analysis in order to ensure amplification and the generation of a single specific gene amplicon. The comparative ΔΔCt method (2^-ΔΔCt^) was used for determining the fold change by averaging the mean values normalized against *GAPDH* and *TBP* reference genes. For each experiment, relative changes in mRNA in experimental samples compared with Ctrl-shRNA taken as 100% were calculated.

##### TH Western blot

The proteins from two sides of the punched LCs were separated by gel electrophoresis (Burnette, 1981). The separated proteins were transferred onto nitrocellulose membranes and blocked with 3% bovine serum albumin in Tris-buffered saline (TBS). Following two washes of 10 min each in TBS containing 0.1% TBS–Tween 20 (TBST) and a single TBS wash, the membrane was incubated with 1:2000 TH primary antibody for 3 h at room temperature with gentle agitation. This was followed by two washes with TBST, and then the membrane was incubated with HRP-linked secondary goat anti-rabbit antibody (Vector Laboratories) for 2 h. The membranes were thoroughly washed three to four times to remove the unbound secondary antibody, and the chemiluminescence of the TH-specific band was detected using Clarity (Bio-Rad). The intensities of TH bands separately in the right and left LC punches were estimated densitometrically using Alpha Imager Software (Alpha Innotech).

##### Immunohistochemistry

To confirm whether the TH-shRNA was effective in downregulating TH in the LC neurons, the brain sections of group III rats containing the bilateral LC were immunostained with TH-Ab using the standard avidin–biotin complex method. Alternate rostrocaudal free-floating coronal sections having both sides of the LC, as per the rat brain atlas (Paxinos and Watson, 1998), were collected for TH immunostaining. The sections were blocked with 0.1 m PBS containing 0.25% Triton X-100 and 10% NGS. Then, the sections were incubated in (1:5000) anti-TH polyclonal Ab raised in rabbit (catalog #AB152, Millipore; RRID:AB_390204) for 72 h at 4°C. After 72 h of incubation, the sections were washed three times with PBS, followed by incubation in biotinylated goat anti-rabbit IgG (catalog #PK-6101, Vector Laboratories; RRID:AB_2336820) for 18 h at 4°C. At room temperature, the sections were washed and treated with the avidin–biotin–peroxidase complex (catalog #PK-6101, Vector Laboratories; RRID:AB_2336820) at 1:50 dilutions for 2 h followed by incubation in diaminobenzidine (chromogen) and hydrogen peroxide (DAB Kit, Vector Laboratories) for 2–6 min until a brown color was observed. Finally, the sections were washed with distilled water; mounted on slides; dehydrated using increasing grades of alcohol; and coverslipped with a mixture of distyrene, tricresyl phosphate (or plasticizer), and xylene mounting medium for microscopic examination and cell counting**.**

Under an Olympus BX51 Microscope, the anatomical extent of the LCs was identified based on landmarks shown in the Paxinos and Watson (1998) atlas. Images of every third section were obtained at 20× magnification. Using Adobe Photoshop, distinct TH-positive neurons within the defined left and right LCs (i.e., the side received TH-shRNA or Ctrl-shRNA) were separately counted in three to five sections from each rat brain. The TH-positive neurons per LC from *n* = 5 rats with Ctrl- or TH-shRNA were averaged separately and compared statistically.

#### Experiment 2: microinjections and sleep recordings

After recovery from surgical trauma and acclimatization, rats in groups IV and V were connected to the recording cables and bipolar EEG, EOG, and EMG recordings were made between 10:00 A.M. and 10:00 P.M. using a Grass Model 7 polygraph following published methods (Mallick et al., 2001; Pal and Mallick, 2009). The recordings were performed under control (without any treatment) and experimental (after treatment as described below) conditions. The electrophysiological signals were also recorded digitally at the sampling rate of 128 Hz using Vital Recording Software and later analyzed manually by SleepSign Software (Kissei Comtec).

##### TH-shRNA microinjection bilaterally into the LC and simultaneous infusion of Saline or NA bilaterally into the PPT

On the day of surgery, 0.5 μl of TH-shRNA was injected bilaterally into the LC of the group IV rats, which also had bilateral guide cannulae aimed at the PPT in addition to EEG, EOG, and EMG electrodes. After postsurgical recovery and habituation, on the first experimental day the sleep–waking recording was done for 12 h after saline (Sal; 0.2 μl) microinjection bilaterally into the PPT. On the next day, the recording was performed during the same period after the PPT was bilaterally infused with 0.2 μl of 0.1 μm NA. In the same rats, sleep–waking recordings were again obtained for 12 h after 3 weeks as postinjection recovery recordings.

Group V rats received Ctrl-shRNA bilaterally during surgery and after 8 d of a postsurgical recovery period, sleep–waking recording was performed for 12 h as described above. The baseline data from rats in group VI were used as the baseline control data for groups IV and V.

##### Microinjection of TH-siRNA bilaterally into the LC and sleep–wakefulness recording

Group VI rats were surgically implanted with guide cannulae aimed at bilateral LC. These chronic rats received either TH-siRNA or Ctrl-siRNA bilaterally in the LC. After postsurgical recovery and acclimatization, baseline and control experiments were conducted on the same animal in group VI rats; thus, the same animal served as its own control. On day 1, the baseline was recorded between 10:00 A.M. and 10:00 P.M. On day 2, 0.5 µl of TH-siRNA was microinjected bilaterally into the LC, and the next day sleep–wakefulness was recorded between 10:00 A.M. and 10:00 P.M. One week later, the same rats received 0.5 µl of Ctrl-siRNA, and sleep–wakefulness was recorded during the same period.

#### Analysis of sleep–wakefulness records

The electrophysiological recordings were first scanned visually to mark and eliminate any obvious disturbance or noise. Following the essential basic criteria [with minor nonessential modifications (e.g., epoch length of 5 s instead of 10 s)], EEG, EMG, and EOG records were manually scored (off-line) in waking state, NREMS, and REMS in bins of 5 s, as reported in detail previously (Mallick et al., 2001). The records were classified as REMS when there was EEG desynchronization accompanied with simultaneous muscle atonia in the EMG and frequent eye movements (unlike in NREMS) in the EOG usually following NREMS (signs of deep sleep). In three group IV rats (i.e., in TH-shRNA-injected rats), the EEG during REMS episodes through the entire 12 h recording period after the injection of saline and NA into the PPT were subjected to spectral analysis using SleepSign software, and the theta power during REMS was evaluated. The total number of recordings under the baseline, upon the microinjection of TH-siRNA/TH-shRNA and Ctrl-siRNA/Ctrl-shRNA bilaterally into LC, were scored in blocks of light phase (10:00 A.M. to 6:00 P.M.) and dark phase (6:00 to 10:00 P.M.). The total time spent in each of those states, the number of episodes of REMS, and the average REMS duration per episode under various conditions were calculated from all the experimental and control rats and was statistically analyzed.

#### Histology

For identification of microinjection sites in the brain, the rats were deeply anesthetized with ketamine (80 mg/kg, i.p.) and xylazine (20 mg/kg, i.p.). To mark the injection sites in PPT and LC, 0.2 μl of 2% pontamine sky blue dye was injected in the same manner as the other injections. The rat brains were intracardially perfused with 100 ml of Sal followed by 4% paraformaldehyde. The brains were extracted, and 40 μm coronal cryosections (Leica) were processed for neutral red staining following published reports (Mallick et al., 2001; Khanday and Mallick, 2015). The microinjection sites were identified by the presence of a bluish stain and were reconstructed by plotting on the corresponding rat brain atlas (Paxinos and Watson, 1998) section. The microinjection of TH-shRNA into LC was confirmed by TH-immunostain, as described above.

#### Experiment 3: REMSD and microinjection of TH-shRNA and TH-siRNA

The REMSD and microinjection studies were conducted in groups VII–XI rats (Table 1). Group VII rats were free-moving control (FMC) rats. Groups VIII and IX had guide cannulae implanted bilaterally aiming the LC for microinjection of Ctrl-siRNA or TH-siRNA, respectively. After recovery from surgical trauma, these rats were deprived of REMS for 24 and 48 h and then microinjected with 0.5 μl of 0.01 nmol either Ctrl-siRNA or TH-siRNA, respectively, bilaterally into the LC and REMSD continued for the next 2 d (i.e., the rats were deprived of REMS for a total of 96 h). The rats in groups X and XI received a single dose of Ctrl-shRNA or TH-shRNA, respectively, bilaterally into the LC during surgery and were allowed 8 d of recovery followed by 96 h of REMSD.

#### Estimation of Na-K ATPase activity

REMS-deprived rats that received a TH-shRNA, TH-siRNA, or control injection were killed using a guillotine. The brains were quickly removed, placed on ice, and homogenized in 10 ml of ice-cold homogenizing buffer containing 0.32 m sucrose; 12 mm Tris, pH 7.4; and 1 mm EDTA. The Na-K ATPase activity in the brain homogenate was estimated following published reports (Gulyani and Mallick, 1993; Amar and Mallick, 2015). The ouabain-sensitive Na-K ATPase activity was estimated and expressed as micromoles of Pi per milligram of protein per hour.

#### Statistical analysis

All data have been presented as the mean ± SEM. Statistical analysis was performed using SigmaPlot version 12.1 (SYSTAT), as shown in Table 3. The mean values from each experimental group (i.e., waking state, NREMS, and REMS) for Na-K ATPase activity and TH-expression in control and experimental rats were statistically compared using ANOVA followed by a pairwise multiple-comparisons (Student–Newman–Keuls *post hoc*) test. The differences in the number of immunostained TH neurons and qRT-PCR data from different groups were statistically compared using Student’s *t* test; a *p* value of at least <0.05 was considered to be significant.

**Table 3:** Statistical table

	Data structure	Type of test	Observed power
a	Normal distribution	Student’s *t* test	1.00
b	Normal distribution	Student’s *t* test	1.00
c	Normal distribution	Student’s *t* test	1.00
d	Normal distribution	One-way ANOVA	0.66
e	Normal distribution	One-way ANOVA	1.00
f	Normal distribution	One-way ANOVA	0.98
g	Normal distribution	One-way ANOVA	0.95
h	Normal distribution	One-way ANOVA	1.00
i	Normal distribution	One-way ANOVA	0.88
j	Normal distribution	One-way ANOVA	0.94
k	Normal distribution	One-way ANOVA	0.91
l	Normal distribution	One-way ANOVA	0.91
m	Normal distribution	One-way ANOVA	0.89
n	Normal distribution	One-way ANOVA	0.66
o	Normal distribution	One-way ANOVA	1.00
p	Normal distribution	One-way ANOVA	0.95
q	Normal distribution	One-way ANOVA	0.98
r	Normal distribution	One-way ANOVA	1.00
s	Normal distribution	One-way ANOVA	0.91
t	Normal distribution	One-way ANOVA	0.89
u	Normal distribution	One-way ANOVA	0.89
v	Normal distribution	One-way RM ANOVA	0.84
w	Normal distribution	One-way RM ANOVA	0.82
x	Normal distribution	One-way RM ANOVA	0.92
y	Normal distribution	One-way RM ANOVA	1.00
z	Normal distribution	One-way ANOVA	1.00
aa	Normal distribution	One-way ANOVA	0.99
ab	Normal distribution	One-way ANOVA	0.91

## Results

### Downregulation of TH mRNA and TH expression in LC-NAergic neurons

#### Downregulation of TH mRNA

**Figure 1.**
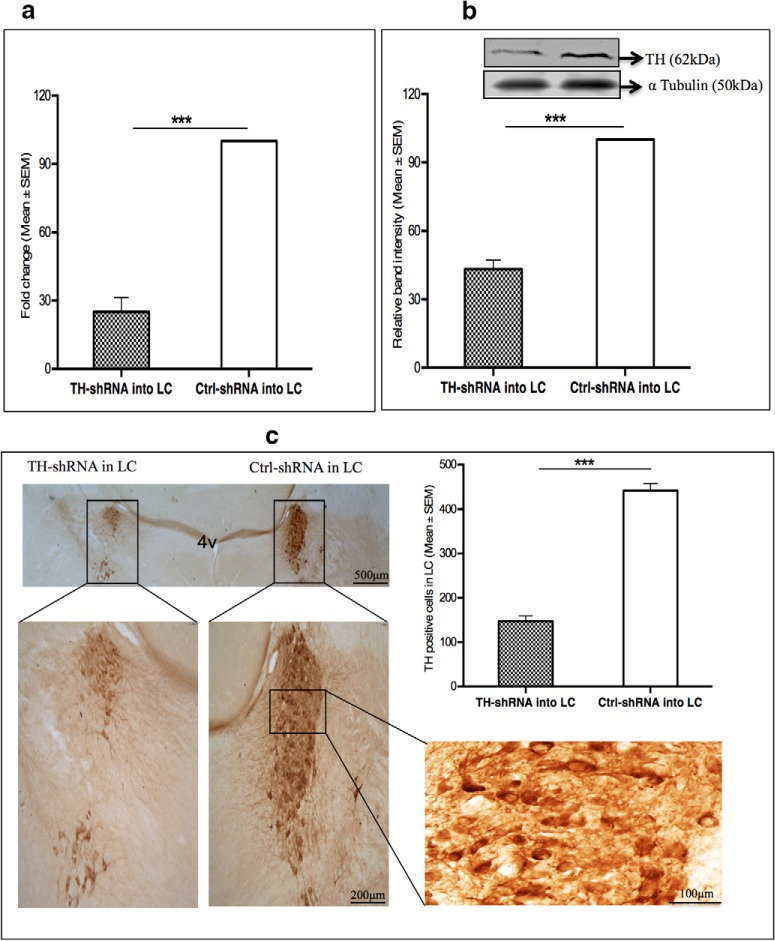
Under stereotaxic surgery, either TH-shRNA or Ctrl-shRNA was microinjected into the right and the left LC, respectively, in the same rat in three groups (I, II, and III). ***a***, ***b***, Eight days after injection, the left and right LCs were punched from group I (*n* = 5) and group II (*n* = 4) rats to estimate TH mRNA (***a***) and TH protein (***b***), respectively. ***c***, Group III (*n* = 5) rat brains were taken out, and sections containing LC were immunostained with anti-TH antibodies. ***a***, For the normalization of TH gene expression, both *GAPDH* and *TBP* were taken as reference genes. Taking the *TH* mRNA expression in the Ctrl-shRNA injected rats as 100%, the percentage change *TH* mRNA expression in TH-shRNA injected rats was estimated. The histogram shows that the TH mRNA was significantly downregulated by ∼74% (***p < 0.001) in the side of the LC that received TH-shRNA compared with the side of the LC that received Ctrl-shRNA. ***b***, Top, Western blot of TH and α-tubulin in samples from punches of LC that received either TH-shRNA or ctrl-shRNA. The TH protein expression was normalized against α-tubulin expression in respective samples and was taken as 100%. The histogram shows that TH protein levels in the LC that received TH-shRNA was reduced by ∼56% (***p < 0.001) compared with the side of LC that received Ctrl-shRNA. ***c***, A representative immunostained section containing LCs is shown in various magnifications (scale bar marked in the figure). The mean (±SEM) count of TH-positive neurons from three alternate sections from each rat (*n* = 5) containing LC that received TH-shRNA or Ctrl-shRNA have been shown in the histogram. There was an ∼66% reduction (***p < 0.001) in the TH-positive neurons in the LC that received TH-shRNA compared with the side of LC that received Ctrl-shRNA.

In group I rats the levels of TH mRNA was significantly (*t*_(8)_ = 11.89, *p* < 0.001)_a_ reduced by ∼74% in the LC, which received TH-shRNA compared with the LC on the other side, which received Ctrl-shRNA (Fig. 1a). The levels of the housekeeping nontargeted α-tubulin mRNA in those LC punches were comparable.

#### Downregulation of TH protein

##### Western blot of TH protein

The TH expression in group II rats was significantly (*t*_(6)_ = 14.47, *p* < 0.001)_b_ reduced by ∼56% in the LC sample from the side that received TH-shRNA compared with the LC on the other side in the same rat, which received Ctrl TH-shRNA (Fig. 1b).

##### Immunohistochemical analysis

In group III rats, one side LC received TH-shRNA, while the other side received Ctrl-shRNA, and after 8 d the brain sections were processed for immunostaining. A representative histological section through LC immunostained with anti-TH antibody is shown in Fig. 1c (left). The TH-positive neurons were significantly (*t*_(8)_ = 14.40, *p* < 0.001)_c_ reduced by ∼66% (Fig. 1c, right histogram) in the LC that received TH-shRNA compared with the side that received the Ctrl-shRNA.

##### Confirmation of localization of the microinjection sites in the LC and the PPT

**Figure 2.**
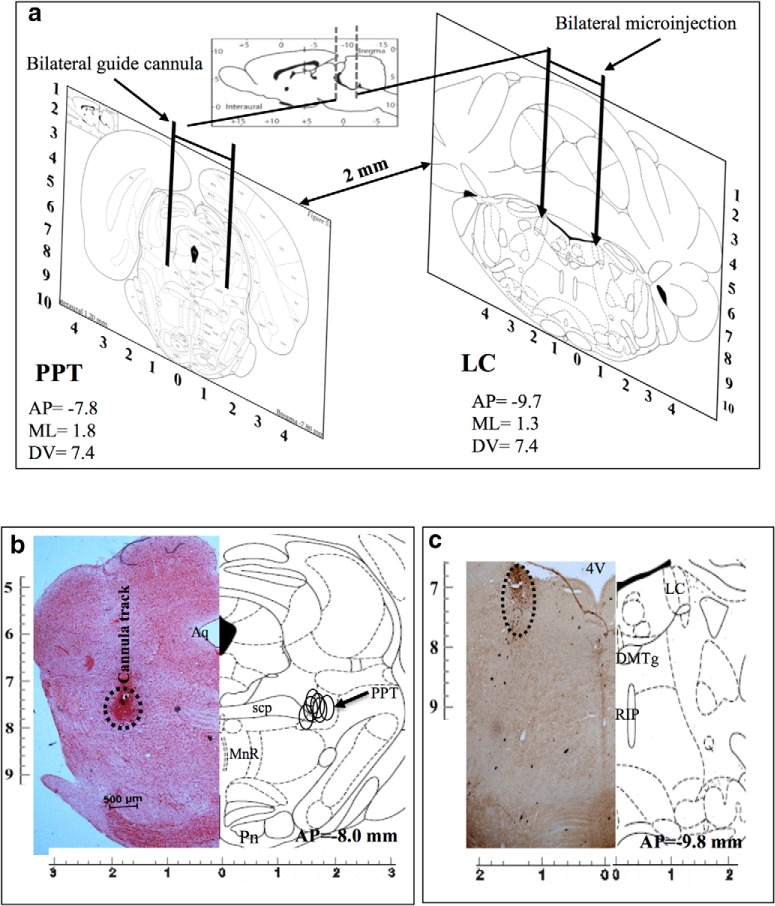
***a***, Diagrammatic representation of rat brain atlas sections through PPT (left) and LC (right) showing implantation of guide cannulae for microinjections into respective sites. ***b***, ***c***, Photomicrographs of representative histological hemisections of experimental rat brains through (***b***) PPT (neutral red stained) and (***c***) LC (TH antibody immunostained) aligned against corresponding rat brain atlas hemisection have been shown. Reconstructed microinjection on-target sites of NA into PPT (*n* = 5) have been shown (open circle) on the right half of the brain atlas hemisection (***b***). As the TH-shRNA was microinjected into the LC during surgery and the injector cannula was removed, it was extremely difficult to trace the extension of the cannula tract on the histological sections. Hence, we confirmed the microinjection site by identifying very few TH-positive neurons in the anatomical LC site (as marked). 4V, 4th ventricle; Aq-, aqueduct; DMTg, dorsomedial tegmental area; MnR, medial raphe nucleus; Pn, pontine nuclei; RIP, raphe interpositus nucleus; scp, superior cerebellar penducle.

Bilateral implantations of cannulae in PPT and LC are shown in Figure 2a (diagrammatic representation). Representative histological hemisections of PPT and LC aligned with respective corresponding hemisections of rat brain atlas sections are shown in Figure 2, b and c, respectively. The microinjection sites (marked on the figure) on the histological sections were identified either by localization of the ends of the cannula tracts and bluish coloration (Fig. 2b) or by a significantly reduced number of TH-positive neurons (Fig. 2c), as the case may be. The microinjection sites from all the experiments have been reconstructed on the atlas section showing that the microinjections were in the PPT (Fig. 2b) and the LC (Fig. 2c). Similarly, sites of microinjection of TH-siRNA into LC were also confirmed and reconstructed. Data from confirmed microinjection sites were processed for analysis.

#### Sleep–waking recording analysis

##### Effects on sleep–wakefulness upon bilateral microinjections of TH-shRNA into the LC and of Sal into the PPT

**Figure 3.**
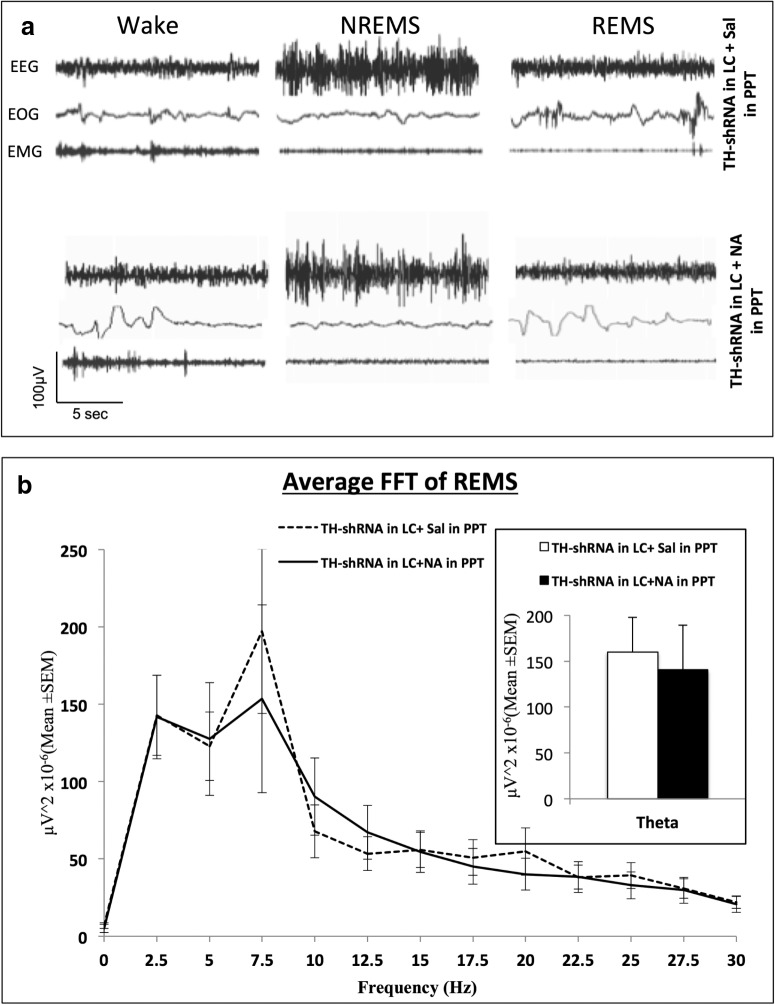
***a***, Representative SleepSign-recorded simultaneous traces of EEG, EOG, and EMG showing waking state, NREMS, and REMS episodes in one rat treated with TH-shRNA injection into LC and injected with saline or NA into the PPT (as labeled on the figure). ***b***, The power spectrum of average FFT (Fast Fourier Transformation) of EEG of all the REMS episodes during the 10:00 A.M. to 10:00 P.M. recording period of one rat under different conditions. Dashed line shows the data of the rat with TH-shRNA injection into LC and saline injection into PPT (total number of episodes of REMS = 131 ± 10), while the continuous line shows TH-shRNA injection into LC and NA injection into PPT (total number of episodes of REMS = 91 ± 13). It shows that the theta waves (5–7.5 Hz) peaked in the background of other frequencies. The histogram in the inset shows the average power of the theta waves (5–7.5 Hz) only of the rats shown in the line plot under the two conditions. It shows that the average power of theta waves was comparable under the two treatment conditions.

*Analysis of 12 h recording.* Representative samples of simultaneous tracings of EEG, EOG, and EMG from one of the rats showing waking state, NREMS, and REMS are shown in Figure 3a. The power spectrum of the 12 h REMS EEG recording of one of the TH-shRNA-treated rats after saline and NA injection into the PPT is shown in Figure 3b. It showed overlapping peaks (after saline and NA injections) at the theta wave frequencies (5–7.5 Hz). Further, the mean (±SEM) power of the theta waves of the same records after saline and NA injections into the PPT was statistically comparable (Fig. 3b, inset).

**Figure 4.**
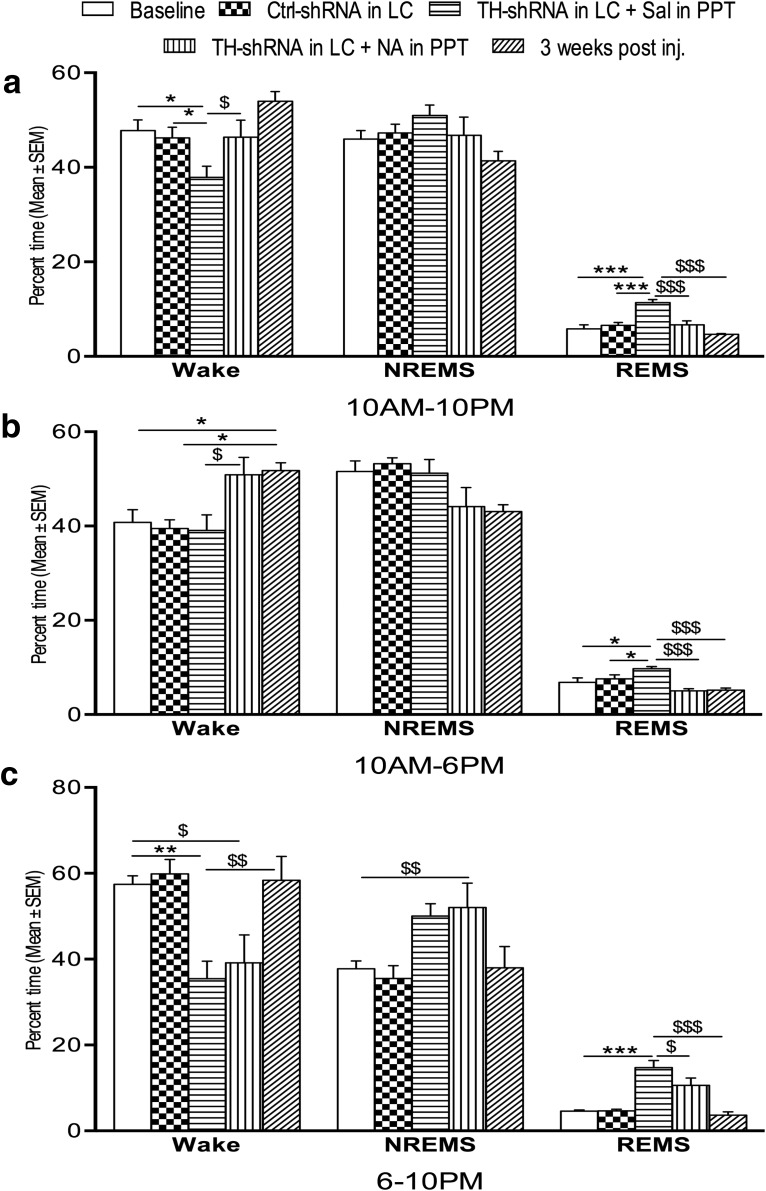
Percentage (±SEM) of total recording time spent in waking state, NREMS, and REMS 8 d after TH-shRNA or Ctrl-shRNA microinjection into the LC and Sal or NA microinjection into the PPT (on the day of recording), and 21 d after TH-shRNA microinjection. ***a–c***, The histograms show analyses of total recording periods of 12 h (10:00 A.M. to 10:00 P.M.; ***a***), 8 h (10:00 A.M. to 6:00 P.M.; ***b***), and 4 h (6:00 to 10:00 P.M.; ***c***). Abbreviations are as in the text. *Compared with baseline values and Ctrl-shRNA injection; $compared with TH-shRNA injection into LC and NA injection into PPT or 3 weeks after the injection of TH-shRNA into LC. Significance level: ***p < 0.001; **p < 0.01; *p < 0.05; *N* = 6.

The percentages of total recording time spent in waking state, NREMS, and REMS during the 12 h (10:00 A.M. to 10:00 P.M.) recording period in the rats infused over the long term with TH-shRNA and Ctrl-shRNA are shown in Figure 4a. The time spent in the waking state (10:00 A.M. to 10:00 P.M.) decreased significantly1 (*F*_(2,15)_ = 5.40, *p* = 0.021)_d_, while the time spent in REMS increased significantly (*F*_(2,15)_ = 17.82; *p* = 0.001)_e_ in the rats that received TH-shRNA bilaterally into the LC and simultaneously Sal into the PPT (group IV) compared with both the rats that received Ctrl-shRNA into the LC (group V) and the baseline rats (without injection, group VI). The percentage of time spent in NREMS in rats from the baseline (control) group was comparable with those rats that received TH-shRNA or Ctrl-shRNA into the LC.

*Analysis of records during the normal light and dark period for rats.* Analysis of data in the first 8 h (10:00 A.M. to 6:00 P.M. light-on period/daytime) of recording is shown in Figure 4b. The percentage of time spent in REMS increased significantly (*F*_(4,25)_ = 7.97, *p* = 0.030)_f_ in the rats that received TH-shRNA into the LC and simultaneously Sal into the PPT compared with the baseline rats and those that received Ctrl-shRNA into the LC and Sal into the PPT. The NREMS and waking states remained comparable in all the rats irrespective of whether microinjections were received or not.

Analysis of the data recorded between 6:00 and 10:00 P.M. (light-off period) is shown in Figure 4c. The percentage of time spent in the waking state decreased significantly (*F*_(4,25)_ = 6.61, *p* < 0.007)_g_, and REMS (*F*_(4,25)_ = 17.60, *p* = 0.001)_h_ increased significantly in rats that received TH-shRNA into the LC and Sal into the PPT compared with baseline and rats that received Ctrl-shRNA into the LC.

The significant changes in sleep–wakefulness after the infusion of TH-shRNA into the LC and Sal into the PPT, as described above, were by and large not seen at 3 weeks after injection (Fig. 4a–c). The rats recovered from the effects induced by TH-shRNA microinjections except for the percentage of rats in the waking state during the period 10:00 A.M. to 6:00 P.M. (incomplete recovery), which remained significantly higher (*F*_(4,25)_ = 5.38, *p* < 0.024)_i_.

**Figure 5.**
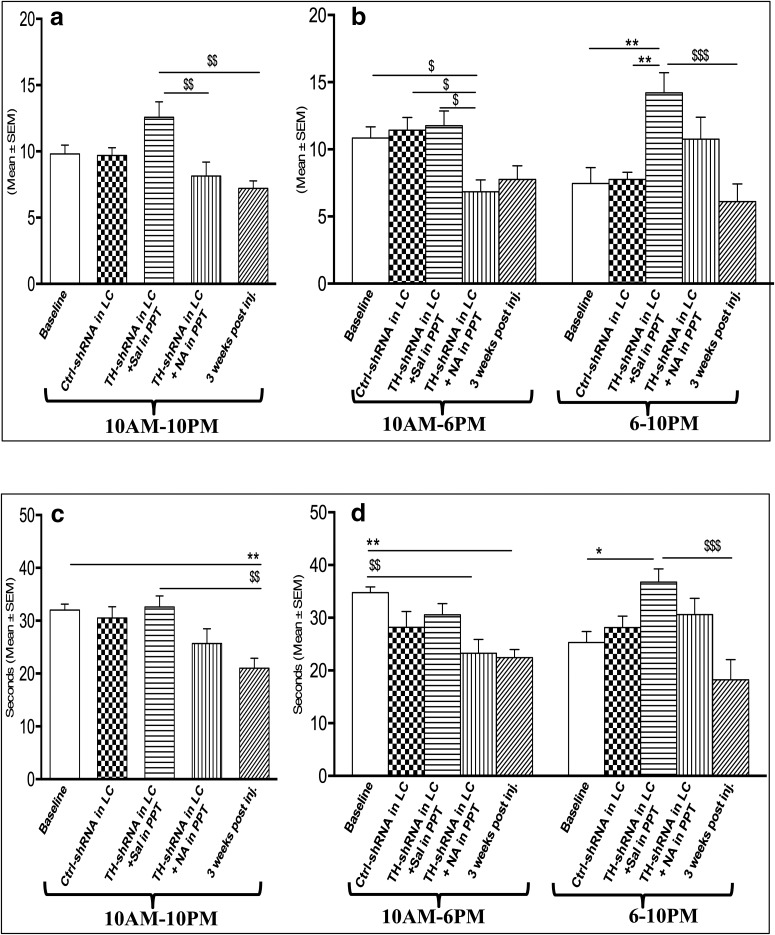
Changes in REMS frequency per hour (top) and REMS duration/episode (s; bottom) during the recording of TH-shRNA or Ctrl-shRNA 8 d after microinjection into LC and Sal or NA microinjection into PPT (on the recording day), and 21 d after TH-shRNA microinjection. ***a–d***, The histograms show analyses for the total recording period of 12 h (10:00 A.M. to 10:00 P.M.; ***a***, ***c***), and 8 h (10:00 A.M. to 6:00 P.M.) and 4 h (6:00 to 10:00 P.M.; ***b***, ***d***). Abbreviations are as in the text. *Compared with baseline and Ctrl-shRNA; $compared with TH-shRNA injection into LC and NA injection into PPT or 3 weeks after the injection of TH-shRNA into the LC. Significance level: ***p < 0.001; **p < 0.01;*p < 0.05; *N* = 6.

*Effects on REMS frequency and duration per episode.* It has been shown above that the duration of REMS was significantly increased in rats that received TH-shRNA into the LC (i.e., in rats where the NA synthesis was downregulated). Therefore, we evaluated whether the frequency (i.e., generation) of REMS per episode, the duration of REMS per episode, or both were affected. The effect of the microinjection of TH-shRNA bilaterally into the LC and Sal into the PPT on REMS frequency per hour during the 12 h (10:00 A.M. to 10:00 P.M.) recording is shown in Figure 5a; while effects during the 8 h (10:00 A.M. to 6:00 P.M.) and 4 h (6:00 to 10:00 P.M.) recording periods are shown in Figure 5b. The REMS frequency per hour during the 10:00 A.M. to 10:00 P.M. and 10:00 A.M. to 6:00 P.M. recording periods did not show significant changes. However, the REMS frequency per hour increased significantly (*F*_(4,25)_ = 6.31, *p* = 0.005)_j_ in the 6:00 to 10:00 P.M. recording block compared with the respective blocks of baseline and Ctrl-shRNA into LC.

The effect of the injection of TH-shRNA bilaterally into the LC and of Sal into the PPT on REMS duration per episode during the 12 h (10:00 A.M. to 10:00 P.M.) recording period is shown in Figure 5c; while data during the 8 h (10:00 A.M. to 6:00 P.M.) and 4 h (6:00 to 10:00 P.M.) recording periods are shown in Figure 5d. The REMS duration per episode in TH-shRNA-injected rats during the 12 h (10:00 A.M. to 10:00 P.M.) recording period was comparable to baseline data and to REMS duration for the Ctrl-shRNA-injected rats. However, the REMS duration per episode in the TH-shRNA-injected rats increased significantly (*F*_(4,25)_ = 5.83, *p* = 0.039)_k_ in the 6:00 to 10:00 P.M. period compared with baseline data and the Ctrl-shRNA-injected rats, while it did not change significantly during the 10:00 A.M. to 6:00 P.M. block.

None of the effects on REMS frequency or REMS duration per episode (6:00 to 10:00 P.M.) upon TH-shRNA injection into the LC described above was observed 3 weeks after injection (i.e., the values returned to normal upon recovery; Fig. 5a,b). However, the effect on the REMS duration per episode during the 10:00 A.M. to 10:00 P.M. (*F*_(4,25)_ = 5.72, *p* = 0.005)_l_ and 10:00 A.M. to 6:00 P.M. (*F*_(4,25)_ = 5.46, *p* = 0.005)_m_ periods did not completely recover to the baseline level at 3 weeks postinjection (Fig. 5c,d).

##### Effects of bilateral microinjections of TH-shRNA into the LC and NA into the PPT

In the same group IV chronically prepared rats (which had received TH-shRNA) presented in the section titled Effects on sleep–wakefulness upon bilateral microinjections of TH-shRNA into the LC 369 and Sal into the PPT, above, on the next day (i.e., after the day of baseline recording) 0.2 μl of NA (instead of Sal) was microinjected bilaterally into the PPT and the effects on sleep–wakefulness recorded. In addition to manual classification of REMS based on classic electrophysiological signals, EEG power spectral analysis of the so-classified REMS showed that the theta power of the EEG after microinjection of saline and NA into the PPT (in those TH-shRNA-treated rats) was comparable (Fig. 3b). The percentage of time spent in the waking state, NREMS, and REMS during the 12 h (10:00 A.M. to 10:00 P.M.) recording have been shown in Figure 4a. The percentage of time spent in the waking state increased significantly (*F*_(2,15)_ = 5.40, *p* = 0.017)_n_, while the percentage of time spent in REMS decreased significantly (*F*_(4,15)_ = 14.41, *p* = 0.001)_o_ compared with that in rats that had TH-shRNA injected into the LC and Sal injected into the PPT. The percentage of time spent in NREMS was comparable to baseline values and to that of Ctrl-shRNA-injected rats (Fig. 4a).

During the 10:00 A.M. to 6:00 P.M. recording, the percentage of time spent in the waking state increased (*F*_(4,25)_ = 5.38, *p* = 0.027)_p_, while that spent in REMS decreased significantly (*F*_(4,25)_ = 7.97, *p* = 0.001)_q_ compared with rats injected with TH-shRNA into the LC and with Sal into the PPT, as shown in Figure 4b. The effect on NREMS was comparable to that in baseline values and Ctrl-shRNA-injected rats. During the 6:00 to 10:00 P.M. recording, the percentage of time spent in REMS also decreased significantly (*F*_(4,25)_ = 17.60, *p* = 0.018)_r_, while the effect on wakefulness and NREMS was comparable to that in rats injected with TH-shRNA into the LC and with Sal injected into the PPT (Fig. 4c).

The effects of TH-shRNA injection into the LC and NA injection into the PPT on REMS frequency per hour during the 10:00 A.M. to 10:00 P.M. recording blocks decreased significantly (Fig. 5a; *F*_(4,25)_ = 5.78, *p* = 0.006)_s_ as well as during the 10:00 A.M. to 6:00 P.M. (Fig. 5b; *F*_(4,25)_ = 5.46, *p* = 0.011)_t_ recording blocks compared with baseline values and rats injected with TH-shRNA into the LC and with Sal into the PPT. During the 10:00 A.M. to 6:00 P.M. recording block, the effects remained comparable to those of the corresponding recording block of rats that received a TH-shRNA injection into the LC and Sal injection into the PPT (Fig. 5b).

The total REMS duration per episode in the rats (group IV) that received TH-shRNA injection into the LC and NA injection into the PPT decreased significantly (Fig. 5d; *F*_(4,25)_ = 5.46, *p* = 0.006)_u_ during the 10:00 A.M. to 6:00 P.M. recording block compared with baseline values, although values remained comparable to those of rats injected with TH-shRNA into the LC and Sal into the PPT during all the recording periods (i.e., 10:00 A.M. to 10:00 P.M., 10:00 A.M. to 6:00 P.M., and 6:00 to 10:00 P.M.; Fig. 5c,d).

##### Effects of microinjection of TH-siRNA bilaterally into the LC on sleep–wakefulness

**Figure 6.**
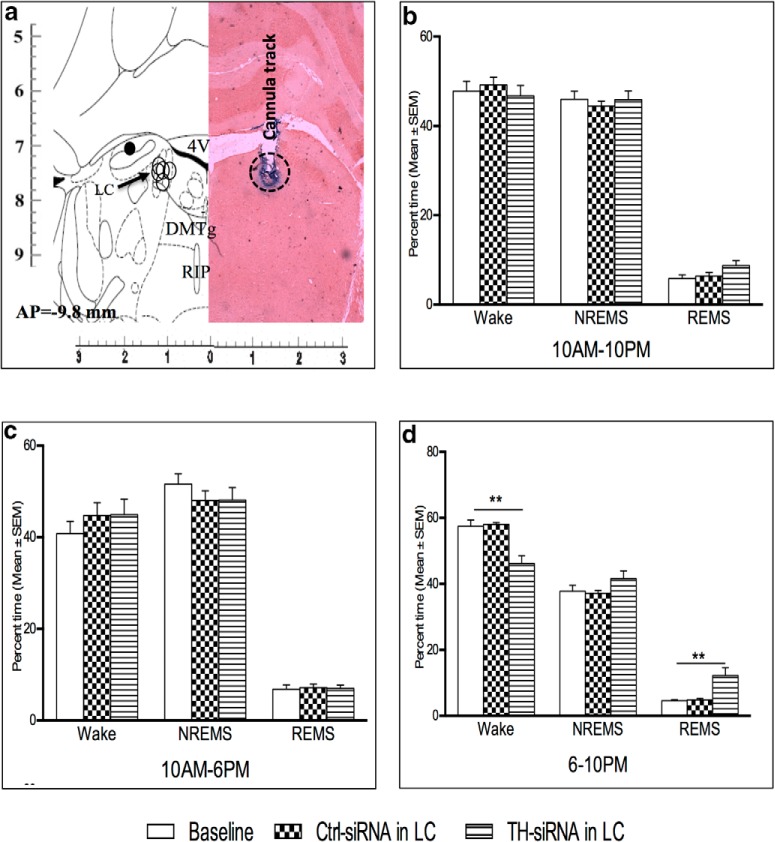
***a***, A representative photomicrograph of a neutral red-stained histological hemisection through LC showing the TH-siRNA microinjection site aligned with the corresponding hemisection of the rat brain atlas is shown. On-target microinjection sites (open circles) from five rat brains have been reconstructed on the left hemisection, which also shows one off-target site (black solid circle). 4V, 4th ventricle; DMTg, dorsomedial tegmental area; RIP, raphe interpositus nucleus. ***b–d***, Percentage (±SEM) of time spent in waking state, NREMS, and REMS 24 h after TH-siRNA or Ctrl-siRNA microinjection into LC during the total recording period of 12 h (10:00 A.M. to 10:00 P.M.; ***b***), 8 h (10:00 A.M. to 6:00 P.M.; ***c***), and 4 h (6:00 to 10:00 P.M.; ***d***) are shown. *Compared with baseline and Ctrl-siRNA values. Significance level: ***p < 0.001; **p < 0.01;*p < 0.05: *N* = 5.

The TH-siRNA injection sites into the LC were histologically confirmed, as detailed above (Fig. 6a). The TH-siRNA was infused bilaterally into the LC in the chronically prepared rats (group VI), and then their sleep–waking state was recorded during the period of 10:00 A.M. to 10:00 P.M. the next day. There was no change in the percentage of time spent in the waking state, NREMS, and REMS in the recording block of 10:00 A.M. to 10:00 P.M. (Fig. 6b) or in block of 10:00 A.M. to 6:00 P.M. (Fig. 6c). However, the TH-siRNA-injected rats spent significantly (Fig. 6d; *F*_(4,14)_ = 9.43, *p* = 0.01)_v_ less time in the waking state, while such injection significantly (Fig. 6d; *F*_(4,14)_ = 8.51, *p* = 0.01)_w_ increased the time spent in REMS during the 6:00 P.M. to 10:00 P.M. block compared with respective baseline (preinjection) values and Ctrl-siRNA-microinjected rats. NREMS was comparable to that of baseline values and those for Ctrl-siRNA-microinjected rats (Fig. 6b–d).

##### Effects on REMS frequency and duration per episode of REMS

**Figure 7.**
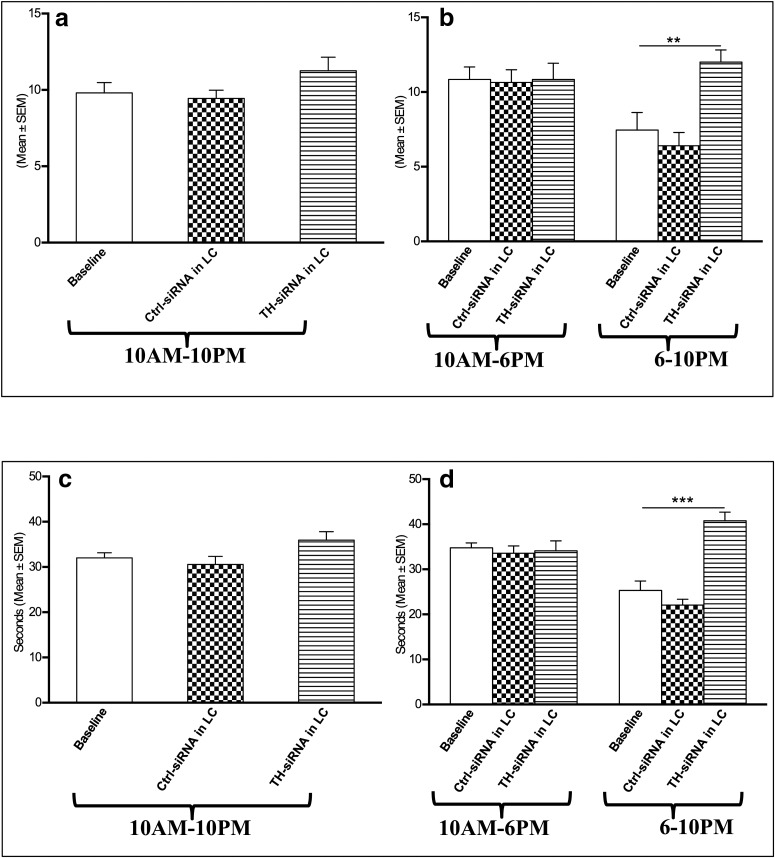
Changes in REMS frequency per hour (top) and REMS duration per episode (s; bottom) 24 h after TH-siRNA or Ctrl-siRNA microinjection into LC. ***a–d***, Analyses for the total period of 12 h (10:00 A.M. to 10:00 P.M.; ***a***, ***c***), 8 h (10:00 A.M. to 6:00 P.M.; ***b***, ***d***), and 4 h (6:00 to 10:00 P.M.; *b*, *d*) are shown. *Compared with baseline and Ctrl-siRNA values. Significance level: ***p < 0.001; **p < 0.01;*p < 0.05; *N* = 5.

There was no change in the REMS frequency per hour either in the recording block of 10:00 A.M. to 10:00 P.M. or in the block of 10:00 A.M. to 6:00 P.M. after TH-siRNA injection, as shown in Figure 7a. Compared with baseline values or those for Ctrl-siRNA injection, the rats that received TH-siRNA showed significantly (*F*_(4,14)_ = 11.12, *p* = 0.005)_x_ increased REMS frequency per hour during the 6:00 to 10:00 P.M. recording (Fig. 7b), which, however, was not significant during the entire 10:00 A.M. to 10:00 P.M. or 10:00 A.M. to 6:00 P.M. daytime recording (Fig. 7a).

The REMS duration per episode also increased significantly (*F*_(4,14)_ = 28.24, *p* = 0.001)_y_ in the 6:00 to 10:00 P.M. block compared with the baseline values and those for Ctrl-siRNA-injected rats. The duration of REMS per episode during the 10:00 A.M. to 10:00 P.M. and 10:00 A.M. to 6:00 P.M. recordings in rats that received TH-siRNA remained comparable to that of baseline values as well as to those of rats that received Ctrl TH-siRNA (Fig. 7c,d).

It is important to note that in one rat where the injection site was off-target to LC (Fig, 6a, filled circle) the above effects on sleep–wakefulness were not seen.

#### Downregulation of TH expression in LC and REMSD studies

##### Effect of REMSD on brain Na-K ATPase activity in rats that received TH-siRNA microinjection bilaterally into the LC

**Figure 8.**
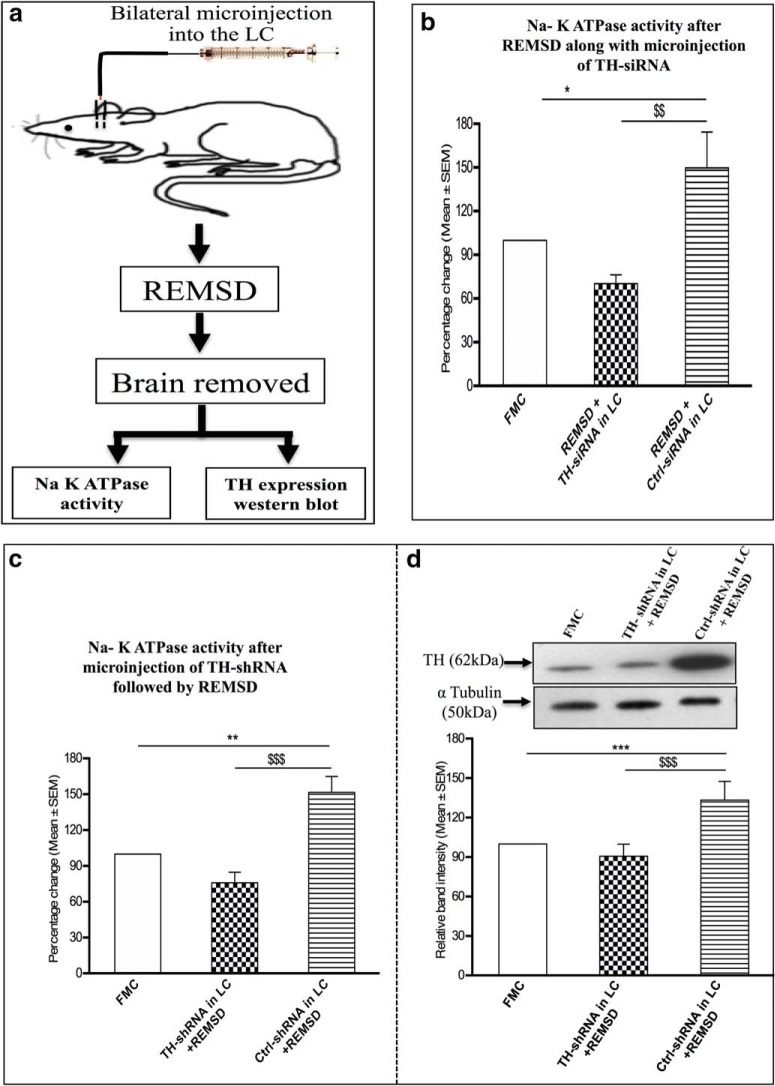
***a***, Diagrammatic representation of protocol of experiment 3. ***b***, Percentage (mean ± SEM) changes in Na-K ATPase activity in the brain homogenate of rats deprived of REMS after microinjection of TH-siRNA or Ctrl-siRNA bilaterally into the LC compared with the Na-K ATPase activity of FMC rats taken as 100%. ***c***, Percentage (mean ± SEM) changes in Na-K ATPase activity in the brain homogenate of rats deprived of REMS after microinjection of TH-shRNA or Ctrl-shRNA bilaterally into the LC compared with Na-K ATPase activity in FMC rats taken as 100%. ***d***, Percentage (mean ± SEM) change in TH protein expression in the brains of rats deprived of REMS for 96 h after the microinjection of TH-shRNA and Ctrl-shRNA bilaterally into the LC relative to FMC control taken as 100% are shown. *Compared with FMC; $compared with Ctrl-shRNA/Ctrl-siRNA in LC and subjected to REMSD. Significance level: ***p < 0.001; **p < 0.01; *p < 0.05: *N* = 5/group.

The specific activity of Na-K ATPase was estimated in the brain homogenate of FMC rats (group VII) that did not receive any microinjection. Rats from groups VIII and IX were surgically prepared with bilateral guide cannulae into the LC; these rats were deprived of REMS for 96 h, as is schematically represented in Figure 8a. At the end of 24 and 48 h of REMSD, these rats received Ctrl-siRNA (group VIII) or TH-siRNA (group IX) bilaterally into the LC and REMSD continued. At the end of 96 h of REMSD, the brains were removed, and Na-K ATPase activity was estimated from brain homogenate. Upon REMSD, the enzyme activity increased significantly (*F*_(2,12)_ = 9.36, *p* = 0.05)_z_ in Ctrl-siRNA-microinjected rats (group VIII) compared with that of the FMC rat brain (Fig. 8b). However, after REMSD, the group IX rats that received TH-siRNA bilaterally into the LC did not show increased enzyme activity (Fig. 8b). In other words, after REMSD, the enzyme activity in the brains of group IX rats was significantly lower compared with that in the group VIII rats that received Ctrl-siRNA (*F*_(2,12)_ = 9.36, *p* = 0.01)_z_.

##### Effect of TH-shRNA microinjection bilaterally into the LC on Na-K ATPase activity in REMS-deprived rats

The Na-K ATPase activity was estimated in the brain homogenate of REMS-deprived rats that received a single injection of Ctrl-shRNA (group X) or TH-shRNA (group XI) bilaterally into the LC (Fig. 8a). The enzyme activity after REMSD increased significantly (*F*_(2,12)_ = 17.34, *p* = 0.01)_aa_ in rats that received Ctrl-shRNA compared with the FMC as well as those that received TH-shRNA (Fig. 8c). However, after REMSD the brain enzyme activity did not increase (*F*_(2,12)_ = 17.34, *p* = 0.001)_aa_ significantly in the rats that received TH-shRNA bilaterally into the LC (Fig. 8c).

##### Effect of TH-shRNA microinjection bilaterally into LC on TH expression after REMSD

The TH expression was estimated by Western blot in the same brain homogenates of rats in groups VII, X, and XI (mentioned above) in which TH-shRNA was injected and Na-K ATPase activity was estimated. Upon REMSD, although the TH expression increased significantly (*F*_(2,12)_ = 82.19, *p* = 0.001)_ab_ in the brains of rats that received Ctrl-shRNA (compared with FMC), the TH expression remained unaffected (comparable to the FMC) in the rats that received TH-shRNA (even though they were deprived of REMS), as shown in Figure 8d.

## Discussion

REMS is an essential physiological phenomenon; it is affected in almost all psychosomatic disorders. Although it may be modulated by many factors, based on isolated independent studies, it has been generally accepted that at the cellular level REMS is regulated by the interaction of NAergic REM-OFF neurons and cholinergic REM-ON neurons (Hobson et al., 1975; Jones, 1991; Crochet and Sakai, 1999; Mallick et al., 2012). Based on isolated independent studies, it was proposed by various groups, including ours, that during NREMS the NA from the NAergic REM-OFF neurons inhibits the REM-ON neurons and the appearance of REMS is prevented. Upon REMSD, the NAergic REM-OFF neurons continue to remain active instead of shutting off (Mallick et al., 1990), resulting in elevated NA levels in the brain, which induce behavioral, cellular, and molecular changes (Mallick and Singh, 2011). However, for the confirmation of isolated findings from independent groups, a complete understanding of the neural regulation of REMS in health and diseases and the application of those findings to patient care, direct evidence of the role of NA released from the LC neurons on PPT neurons in REMS regulation and for amelioration of REMSD-associated symptoms, particularly *in vivo*, was glaringly missing, which has been investigated in this study.

The crucial enzyme necessary for the synthesis of NA is the TH. In this study, TH was downregulated in separate groups of rats by microinjection of lentivirus-coated TH-shRNA or TH-siRNA into the rat LC *in vivo*. The downregulation of TH was confirmed (1) by estimating the TH mRNA level by qRT-PCR; (2) by estimating the TH protein level by Western blot in the punched LC (the site of Ctrl-shRNA or TH-shRNA microinjection); and (3) by counting the number of TH-immunostained neurons in the LC. The downregulation was specific because the TH expression was significantly reduced in the punched LC, which received TH-shRNA, and not in the side of the LC, which received Ctrl-shRNA. Thus, the same rat served as its own control and ruled out nonspecific effects, if any. The downregulation of TH in the neurons reduces NA synthesis and release into projected areas. In this study, we have observed that the downregulation of TH in the LC caused increased REMS, which was overcome by the microinjection of NA into the PPT, the site of REM-ON neurons. Further, upon REMSD the Na-K ATPase activity did not increase in the TH-downregulated rat brains, although it remained elevated in the REMS-deprived rat brains, which had received Ctrl-siRNA or Ctrl-shRNA. The detailed findings of this study have been discussed below with perspectives under three subheadings.

### Knockdown of NA synthesizing enzyme TH in LC

Application of the RNAi is a fascinating tool to reversibly downregulate the expression of a desired protein. For exploring the functions of a gene, the use of classic approaches of generating mutants or transgenics are not only time consuming, laborious, and less specific, but also more expensive than silencing genes by using RNAi. Additionally, compared with mutant or transgenic animals, gene silencing using RNAi technology is a reversible process, and, therefore, recovery can also be studied. In support, in this study we observed that the neurons were not permanently damaged because most of the effects of treatment of TH-shRNA returned to a normal level after 3 weeks (recovery); those effects that recovered partially possibly needed some more recovery time. Previous experiments where the LC neuronal behavior was manipulated in wild-type animals, electrical stimulation, electrical or chemical lesions, or pharmacological interventions were used that did not allow selective and reversible manipulations of LC-NA neurons. In this study, the use of virally delivered TH-shRNA enabled us specifically and effectively to target NAergic neurons in the LC, avoiding other adjacent neurons as well as fibers of passage. In addition, this approach avoided developmental and adaptive changes that could alter endogenous circuits, which is also a drawback of the transgenic models. Thus, in the present study the use of TH-shRNA and TH-siRNA overcame the limitations described above. It knocked down TH, the rate-limiting enzyme of NA synthesis, and evaluated its impact on REMS regulation and functions, more importantly *in vivo* in rats where most of the REMS-related studies have been reported.

It was observed that TH-shRNA injection into the LC significantly reduced the expressions of both the TH mRNA as well as the TH protein; the number of TH-expressing neurons was also significantly reduced compared with that of the Ctrl-shRNA injection level. This reduction in TH expression caused increased REMS and ameliorated REMSD-induced effects at the molecular level. The increased REMS was due to an increase in both the REMS duration per episode as well as the REMS frequency of occurrence. The results of this study may be supported by those of previous studies in which the inactivation of LC neurons either by local cooling (Cespuglio et al., 1982) or by chemical withdrawal of inhibition (Mallick et al., 2001; Chen et al., 2010) increased REMS, while the activation of LC neurons reduced REMS (Singh and Mallick, 1996; Kaur et al., 2001; Carter et al., 2010; Jaiswal et al., 2010).

Both TH-siRNA as well as TH-shRNA injection into the LC increased REMS and prevented an REMSD-associated increase in Na-K ATPase activity. However, a closer look at the data shows that, although the effect of TH-shRNA on REMS was (statistically) significant for the entire 12 h recording period, the effect of TH-siRNA on REMS was (statistically) significant during the dark period when the rats, being nocturnal, remain awake. This may be attributed to the fact that the knock-down efficiency of TH-shRNA is reported to be better than that of TH-siRNA (Rao et al., 2009). However, one may argue that TH-siRNA and TH-shRNA were almost equally effective on Na-K ATPase activity in the brain. This could be due to the fact that the Na-K ATPase activity was not estimated at varying time points and more importantly, unlike the relatively instantaneous changes in electrophysiological signals used to classify REMS, the biochemical and molecular changes, once induced, take a longer time to recover. As a critical analysis, we should also highlight that our rats were maintained on a 12 h light/dark cycle with lights on at 7:00 A.M. However, our dark period analysis was performed for 6:00 to 10:00 P.M. recordings instead of 7:00 to 10:00 P.M. recordings. We did this consciously to avoid the transition effect, if any, due to sudden darkness upon switching off the light. However, whether that has any bearing on our results discussed above remains unanswered. Our findings suggest that downregulation of the TH expression reduced NA release in the projected sites, causing enhanced REMS. This may be supported by earlier isolated findings that levels of TH (Basheer et al., 1998; Majumdar and Mallick, 2003; Somarajan et al., 2016) and NA (Porkka-Heiskanen et al., 1995; Mallick and Singh, 2011) were elevated upon REMSD, while the NA level in the brain has been reported to be reduced during REMS (Léna et al., 2005; Gottesmann, 2011).

### LC NAergic inputs to PPT prevent appearance of REMS, advancing our knowledge on the neural mechanism of why REMS normally does not appear during waking or NREMS period

Independent (Hobson et al., 1975) as well as simultaneous (Mallick et al., 1998) recording of LC NAergic REM-OFF neurons and PPT REM-ON neurons led to proposal of the existence of a reciprocal relationship among those REM-ON and REM-OFF neurons. Subsequently, findings from isolated and independent studies led us to propose the crucial role of NA and GABA on PPT neurons leading to the generation of REMS (Mallick et al., 2001; Pal and Mallick, 2004, 2006). Based on such isolated independent studies, we postulated that the NA from the REM-OFF neurons in the LC inhibit the REM-ON neurons in the PPT to prevent the generation of REMS and that this inhibition is withdrawn for the generation of REMS (Mallick et al., 2012). These neural connections have been mathematically modeled, and the modeling revealed that neither the inhibition of the NAergic REM-OFF neurons nor the stimulation of the REM-ON neurons alone would induce REMS; rather, both have to be executed simultaneously (Kumar et al., 2012), which is extremely difficult to demonstrate experimentally, particularly by conducting *in vivo* studies. Thereafter, recent findings from the stimulation of PPT neurons by the application of recently developed optogenetic technology confirmed a role of cholinergic PPT neurons in REMS regulation (Van Dort et al., 2015). However, direct evidence of an inhibitory role of NA from LC neurons in preventing generation of REMS and whether the NA acted on the PPT neurons preventing the appearance of REMS is still unknown. Also, it is unclear, particularly *in vivo*, whether the increased NA released from the LC neurons was actually responsible for REMSD-associated molecular changes.

Our results showed that the downregulation of TH, which inhibits the synthesis of NA, increased REMS and this effect was withdrawn if NA was infused into the PPT. Thus, this study confirms *in vivo* that NA from LC neurons inhibits PPT neurons and prevents REMS; this explains why REMS normally does not appear during waking. The findings are also consistent with those of previous reports that LC REM-OFF neurons continue to remain active during REMSD (Mallick et al., 1990), activation of LC neurons prevents the appearance of REMS (Singh and Mallick, 1996; Mallick et al., 2012), *in vitro* NA inhibited cholinergic neurons in the PPT (Mühlethaler and Serafin, 1990), and intraperitoneal injection of NA agonist (Leppävuori and Putkonen, 1980) and systemic application NA reuptake blocker (Ross et al., 1990) decreased REMS (Tononi et al., 1991; Cirelli et al., 1992). Also, these results may be supported by earlier reports that REMS was increased by reducing the NA release in the PPT by infusing the α2 presynaptic autoreceptor agonist clonidine or by preventing NA to act on PPT neurons by infusing the α1 adrenergic antagonist prazosin (Pal and Mallick, 2006). The results are site specific because if the microinjections did not hit the target site, the effects were not seen. The results of this study are the first direct evidence particularly from *in vivo* studies supporting our proposed neural mechanism of REMS regulation that normally NA from the LC NAergic REM-OFF neurons tonically inhibits the PPT cholinergic REM-ON neurons and prevents the appearance of REMS. Further, the downregulation of NA in the LC neurons disinhibits (activates) the PPT REM-ON neurons, and REMS is actively initiated (Mallick et al., 2012).

### Elevated NA level from LC neurons induces REMSD-associated symptoms

Independent studies have reported that NA levels may rise as a result of REMSD, while elevated levels of NA may also induce REMS loss. Because of the complex nature of REMS, these events may not necessarily always be strictly temporally correlated or have a sequential causal relationship. We observed that the increased release of NA from the LC REM-OFF neurons is responsible for REMSD-associated symptoms. REMS is a unique phenomenon, and its loss affects almost all physiological and behavioral processes; however, the detailed underlying mechanism of inducing REMSD-associated changes was not yet known. As a unified hypothesis, it was proposed that REMS maintains brain excitability (Mallick et al., 1994) and, thus, serves the housekeeping function of the brain; also, many if not most of the effects of REMSD are somehow mediated by an elevated level of NA in the brain (Mallick and Singh, 2011). An estimate of the Na-K ATPase activity was taken as a reflection of the proportionate change in brain excitability, and its regulation by NA has been studied extensively (Mallick and Singh, 2011; Amar and Mallick, 2015). As the NAergic neurons in the LC normally cease firing during REMS but continue activity during REMSD (Mallick et al., 1990), we had postulated that upon REMSD the level of NA increases in the brain due to its increased synthesis and increased release (due to continuous activity of the LC neurons). However, the hypothesis was based on findings from isolated studies and direct evidence, particularly from *in vivo* studies, was lacking. It needed to be confirmed *in vivo* that the LC neurons are the source of NA for inducing REMSD-associated symptoms.

Therefore, to confirm in this study that different groups of experimental rats were deprived of REMS following the downregulation of TH mRNA as well as TH protein in the LC neurons by injecting TH-shRNA or TH-siRNA bilaterally into the LC and Na-K ATPase activity was estimated in the brain samples; suitable control studies were also conducted to rule out nonspecific effects. We observed that, upon REMSD, the Na-K ATPase activity was increased in the brains of the rats that received Ctrl-shRNA injection to a level that was comparable to that of the already reported upon REMSD of otherwise normal cage control rats (Gulyani and Mallick, 1993). However, upon REMSD the enzyme activity failed to increase in the rat brains that received either TH-shRNA or TH-siRNA injections into the LC. Earlier studies have reported that under similar condition the Na-K ATPase activity was increased in the rat brains after REMSD, and the effect was mediated by NA (Mallick and Singh, 2011; Amar and Mallick, 2015; Somarajan et al., 2016). The NA-induced effect, activation of the enzyme in the whole brain in this case, may be supported by the fact that the LC neurons have been reported to project throughout the brain (Samuels and Szabadi, 2008), and increased NA as well as TH mRNA have been reported in the rat brain after REMSD (Porkka-Heiskanen et al., 1995; Basheer et al., 1998). Also, it has been reported that in rat brains REMSD-induced changes in several enzyme activities (Thakkar and Mallick, 1991, 1993a,b; Amar and Mallick, 2015) and neuronal cytomorphology (Ranjan et al., 2010) are mediated by elevated level of NA.

This is the first direct evidence *in vivo* that the REMSD-induced effects are mediated by the NA released from the LC NAergic REM-OFF neurons. This is most likely because upon REMSD the TH is reported to be upregulated in the brain (Basheer et al., 1998) and in the LC neurons (Majumdar and Mallick, 2003), which would increase the synthesis of NA. Further, as the LC NAergic neurons continue firing without cessation of their activities, it would increase the release of NA. Notably, upon the downregulation of TH in LC neurons the Na-K ATPase activity did not increase in the rat brain even after REMSD. In support, in a complementary study in rats recently we have shown that REMSD-associated neurodegeneration and apoptosis of neurons were also inhibited if TH was downregulated in the LC neurons of rats deprived of REMS (Somarajan et al., 2016). As Na-K ATPase is the key factor for the maintenance of neuronal excitability, we propose that reduced synthesis of NA in the LC neurons would prevent REMSD-associated alteration in brain excitability, which is expected to prevent REMSD-associated symptoms. Although the findings are in rats, they are proof of principle and would serve as the basis for future studies, including attempting to use it for treating at least those patients experiencing REMS loss and associated symptoms. For example, subject to confirmation using a targeted delivery approach, attempts may be initiated to downregulate the TH in LC NAergic neurons to provide at least some relief to at least select patients experiencing REMS loss, which is associated with some symptoms like elevated NA level, irritability, hyperexcitability, hypothermia, hypertension, and apoptosis. However, as the neural regulation of behaviors is very complex, adjusting the dose and duration of treatment needs to be carefully regulated, which quite understandably would differ from one individual to another and with or without having other comorbidities.

## Synthesis

Reviewing Editor: Quentin Pittman, University of Calgary Decisions are customarily a result of the Reviewing Editor and the peer reviewers coming together and discussing their recommendations until a consensus is reached. When revisions are invited, a fact-based synthesis statement explaining their decision and outlining what is needed to prepare a revision will be listed below. The following reviewer(s) agreed to reveal their identity: James M O'Donnell, Robert Greene.

This is a proof-of-principle study showing some direct evidence in live rats for the role of noradrenaline (NA) on rapid eye movement sleep (REMS), as well as involved neural circuits. Data suggested that reduced NA levels in locus coeruleus (LC) dis-inhibited the activity of REM-ON neurons in the pedunculo-pontine tegmentum (PPT), and therefore resulted in increased REMS. Moreover, the molecular changes associated with REMs deprivation were also reversed by tyrosine hydroxylase knockdown in the LC. The reviewers and I agree that this is a demonstration of 1) the necessity of LC-NE for normal constraint of REM sleep expression and 2) a re-confirmation of the sufficiency of locally applied NE into the PPT to reduce REM sleep. In addition you also show that reducing LC TH prevents one REM sleep deprivation-associated change, increased Na-K ATPase expression .Taken together, it is consistent with a model of LC-NE acting in the PPT to physiologically constrain REM sleep.

The study is technically sound; however, the reviewers have raised the following concerns:

Major:

1. The authors seemingly conflate REM generation with REM sleep intrusion into waking or NREM. REM sleep intrusion into waking or NREM sleep is typically understood as the occurrence of REM-like behavior (generally loss of muscle tone) that appears spontaneously within a waking or NREM bout, for example, a waking to REM transition as is seen in narcolepsy or a RBD-like episode during NREM as can be seen in Lewy Body dementia. It is unclear here whether intra-PPT NA administration alters the occurrence or appearance of REM as opposed to the amount of REM sleep. Are there direct waking to REM transitions with these manipulations? Are there any changes in spectral power (particularly theta), muscle tone, or phasic activity during REM sleep in response to intra-PPT NA? It would also be helpful to explicitly state the criteria that were used to define an epoch as REM sleep and whether the scoring was performed manually or automatically. It is absolutely necessary to determine whether the intra-PPT NA administration results in same EEG, EMG, EOG characteristics as is seen under baseline conditions (i.e. REM sleep as opposed to REM-like sleep) in order to interpret these findings as REM generation or REM sleep intrusion into waking or NREM. This is an important distinction because REM generation implies a global, unified brain state, while REM intrusion into waking or NREM sleep is a state dissociation. Unified vs dissociated states are likely to have different functional consequences.

2. Please clarify the design of the sleep/waking analysis (groups IV-VI). In particular, the 3 week post injection 12 hr recording is mentioned in table 1 and the results section, but I could not find any description of this in the methods. Also, "baseline" in comparison with TH-shRNA and Ctrl-shRNA in LC rats needs to be explicitly explained since neither of these groups has a baseline condition. This baseline is referred to as coming from "baseline rats"; please restate this as the baseline, pre-injection condition from group VI rats (or something to that effect). Furthermore, this baseline condition is used for two different comparisons (vs group IV and V) and (vs other conditions of group VI) and thus the statistics used for each comparison should be different since one is between groups and one is within group, but it does not appear that a repeated measure ANOVA was used for the within group comparison.

3. There are concerns about some of the figures:

Fig 1. All photomicrographs must have a properly labeled scale calibration bar (100 m, 50 m etc) ; 10 x, 20 x etc is not a scale

Fig 2 &amp; 5 use diagrams from a published brain atlas; please ensure that it is cited and that you have permission to reproduce these brain section figures

Fig 7d- the western appears to show bands taken from two different gels with different protein loading. The alpha tubulin and TH bands must be from the same gel.

4. It is stated that the REM sleep time, episode duration, and episode frequency changes seen in group IV are not present during the 3 week post injection recording; however, based on figures 3 and 4, REM frequency and REM duration/episode during the 3 week recovery condition look reduced compared to control and Ctrl-shRNA conditions (figure 4). Also, waking during recovery seems to be increased (figure 3). Also for the experiment shown in figure 3, a control of cntrl-shRNAinLC+NAinPPT is needed.

Minor concerns:

5. In comparing TH-siRNA vs TH-shRNA, there appears to be a greater REM sleep effect with TH-shRNA compared to TH-siRNA, however, they seem to have equivalent effects on Na-K ATPase activity following REM deprivation. The authors attribute the differences in REM sleep outcome on TH-shRNA being long lasting with better knockdown efficiency, but there is no explanation of the relative lack of difference in their molecular REMSD-associated symptom.

6. Throughout the manuscript, the term "TH-siRNA" and "TH-shRNA" were used, however "TH" was never defined. Please provide full spelling whenever an abbreviation appears initially.

7. As the authors stated, not only the NA level in the brain was responsible for loss of REMS, in reciprocal, REMS deprivation also contributed to sustained high levels of NA. While the exact order of events occurring or working mechanism may not simply be one leading to another, the present data was only sufficient in proving the REMS as the downstream effector of NA. This should be made clear.

8. The authors suggested that reducing NA levels in LC might serve as a potential treatment for REMSD-associated symptoms. It would be helpful if even a short list of symptoms be elaborated in the discussion. The main reason for that is because simple manipulation of NA levels in the brain (especially LC) might lead to unexpected outcome such as those related to cognitive performances. For example, adrenergic enhancers or blockers have both been implied as beneficial to Alzheimer's patients. Therefore down regulation of NA in LC in the hope of treat REMSD-associated symptoms may only be applicable in specific scenarios.

Thus the potential clinical relevance should be toned down.

9. The majority of the sleep-waking behavior after 3 weeks post-injection returned back to baseline as the authors indicated, except for the "wake" percent during 10AM-6PM.

10. Page 17, line 406, "groups VII and IX rats" --> "groups VIII and IX"; line 409, "group VII" --> "groups VIII".

11. There are a couple of points related to REM sleep-loss associated phenomena and the potential to treat patients that seem overstated. 1) The authors conclude that reducing TH in the LC prevents REM sleep-loss associated symptoms and behavioral changes on the basis of a single molecular measure (Na-K ATPase). It is unknown whether intra-LC TH reduction prevents REMD-induced LC cellular damage or behavioral changes since neither was measured. This is particularly important since REM sleep time and Na-K ATPase activity seem to be at least partially independent (as discussed below) 2) This manuscript does provide proof of principal as to how REM sleep could be modified, but the suggestion of potential to treat patients seems unreasonable at this point given that the experimental design involved direct administration into the brainstem. Additionally, the REM sleep effects were temporary, by design, so it is unknown whether REM sleep could be persistently changed as would likely be necessary for therapeutic purposes.

12.This manuscript should be carefully edited. Currently there are a number of oddly structured sentences (including the title) that make it difficult to read, a number of typos (for example prepaid instead of prepared), and some inconsistencies (12:12 cycle with lights on at 7am in 'animals' section, but 6pm-10pm used for dark phase analysis, and 'animals were allowed to recover for at least a week' when acclimation including mock injections started on the 4th recovery day, 'one rat microinjection attempted into LC was off target', elsewhere 'it is important to note that in those two rats where the injection sites were off target'). In the significance section: "it has long been proposed" instead of "since long"; please remove 'mother of several disorders"

13. Given that EEG/EMG was not measured during REM sleep dep, please provide some evidence that the combination of animal size to platform size used in the current experiment results in REM deprivation (a citation is sufficient).
